# Diverse Interactions: Root-Nodule Formation and Herb-Layer Composition in Black Locust (*Robinia pseudoacacia*) Stands

**DOI:** 10.3390/plants12183253

**Published:** 2023-09-13

**Authors:** Ágnes Csiszár, Dániel Winkler, Dénes Bartha, Gergely Zagyvai

**Affiliations:** 1 Institute of Environmental Protection and Nature Conservation, 9400 Sopron, Hungary; bartha.denes@uni-sopron.hu (D.B.); zagyvai.gergely@uni-sopron.hu (G.Z.); 2 Institute of Wildlife Biology and Management, University of Sopron, 9400 Sopron, Hungary; winkler.daniel@uni-sopron.hu

**Keywords:** biological indication, invasive tree species, diversity, nitrogen fixation, root-nodule

## Abstract

The black locust (*Robinia pseudoacacia* L.) is the second-most abundant deciduous tree in forest plantations, and one of the most important invasive woody species worldwide. The species has a strong transformer capacity, especially expressed by its nitrogen enrichment effect caused by nitrogen-fixing bacteria living in its root-nodules. The aim of this study was to explore the mutually interacting factors of nitrogen-fixing root-nodules, site characteristics, and herb-layer composition of 28 North Hungarian black locust stands. In the herb-layers of the study sites, a total of 121 plant species were identified, representing a relatively low species richness. The studied black locust stands showed high variability both in their herb-layer compositions and root-nodule formation, but no clear relationship could be demonstrated between these characteristics. The PCA component with which the species richness and Shannon–Wiener diversity index were strongly correlated was negatively associated with all root-nodule parameters (number, surface area, and weight), supporting the biodiversity-reducing effect of black locust by its nitrogen-fixing bacteria. All of the root-nodule parameters were negatively correlated with the PCA factor predominantly determined by stand age, confirming that the root-nodule biomass decreases as time progresses.

## 1. Introduction

The North American black locust (*Robinia pseudoacacia* L.) is the second-most abundant deciduous tree in forest plantations in the world, and is one of the most important invasive woody species worldwide [1]. This species has spread widely throughout Europe since the 17–18th century, and its intense range expansion is expected to continue eastward, especially in northeastern Europe in the near future [2]. A significant part of European black locust stands is concentrated in Hungary. Black locust plantations have become considerable in Hungary since the second part of the 19th century. Currently, 24% of the Hungarian forest stands are covered by black locust, and these trees are also often planted in settlements [3]. The species can be successfully planted in a wide range of habitats, even on the disturbed, heavily polluted soils of urban or industrial areas, although its spread in flooded, oxygen-poor environments is highly constrained [4,5]. In forest ecosystems, the role of vegetative spread by its root suckers is more important, while propagation of its seeds in more open areas can also play a role [6]. Black locust has high importance in wood and honey production in Hungary; therefore, this adventive species is the most common tree in the country. As a successful invader and transformer species [7,8], the black locust has a strong transformer capacity, especially expressed by its nitrogen enrichment effect and allelopathic potential [9,10]. Through litter decomposition, releasing allelopathic substances may play a role in its invasive success [9]. Black locust has a complex effect on soil characteristics, such as increasing the soil’s cation exchange capacity, aggregate stability, water holding capacity, organic matter, extractable K, organic carbon, total nitrogen, and nitrate content [11,12,13,14,15,16,17,18].

Nitrogen fixation is a considerable characteristic of black locust, which has great importance to forestry and nature conservation as well. Planting species that are symbiotic with nitrogen-fixing bacteria could be advantageous in many respects because these species tolerate low nutrient conditions, grow quickly, and have been useful in the stabilization of nutrient-poor soil [19]. Otherwise, the nitrogen availability produced by these species has a strong effect on the soil characteristics, and may transform the plant community composition considerably [20,21]. Some of the most important invasive species of natural ecosystems are nitrogen-fixing, although the percentage of nitrogen-fixing invaders varies considerably in different regions of the world [22,23]. Nitrogen enrichment could further enhance the invasibility of affected ecosystems, since the invaders usually benefit more from higher nutrient availability than the native species [19,24,25].

Most of the nitrogen demand from black locust is gained by *Rhizobium, Mesorhizobium,* and *Bradyrhizobium* bacteria, especially at a young age [26]. Black locust has a wide variety of nitrogen-fixing bacteria [1,27,28,29,30]; therefore, its cultivation and invasive spread are not limited by available symbiotic microorganisms. Bokor’s study [31] proved there is a positive correlation between the number of nodules and the growing strength of trees. No nodule was found under black locust stands under weak conditions, but 500–600 nodules were documented in good stands on sandy soil. Nitrogen fixation is generally the most intensive in the upper soil layer, since the number of root-nodules decreases continuously with depth. The estimated symbiotic nitrogen fixation rates can vary widely from 23 to 300 kg/ha/year [1,32,33,34], while nitrogen enrichment can achieve 100–300 kg/ha in 3–4-year-old black locust stands [3].

The fixed nitrogen is transformed rapidly into available forms for other plant species through the decaying leaf litter; the direct exudation of nitrogen by black locust roots is not considerable [35]. This nitrogen enrichment leads to rapid and permanent changes in the vegetation and in plant–animal interactions [36,37]. The unification effect of black locust is obvious for the canopy layer, but is of less consequence in the case of the herb-layer. The herb-layer composition of black locust stands was studied by several authors within the native and synanthropic areas as well [3,38,39,40,41]. Most studies draw attention to the decrease in species richness and diversity and the dominance of shade-tolerant and nitrophilous species [1,3,42], while some authors highlight the sparse or even lacking vegetation under the black locust canopy [42]. Studying different Hungarian and north Italian *Robinia* stands, Tobisch et al. [38] observed a high similarity of species dominance in Hungarian black locust stands. However, contrary to expectations, the Hungarian and Italian herb-layer composition did not differ significantly. Cierjacks et al. [1] summarized the *R. pseudoacacia* communities, drawing attention to the difference in species assemblages between regions and, therefore, the difficulty of their classification and unification.

As the mentioned literature show, root-nodule formation, nitrogen fixation, and herb-layer composition in black locust plantations could be considerably connected to each other. The aim of this research was to study the development of nitrogen-fixing root-nodules, the site characteristics, and the herb-layer composition of black locust stands as mutually interacting factors. During our research, we sought answers to the following questions: i. How much heterogeneity do the studied stands show in terms of herb-layer composition and root-nodule formation? ii. Is there any correlation between site characteristics and herb-layer composition or between site characteristics and root-nodule formation? iii. Does the herb-layer composition, especially the number and cover of nitrophilous species, indicate the root-nodule parameters (number, surface area, weight)?

## 2. Results

### 2.1. Herb-Layer Composition

In the herb-layer of the study plots, a total of 121 plant species were identified (Table S1), representing a relatively low species richness. The most frequent species (recorded in at least 20 plots of the 28) were the following: *Chelidonium majus, Urtica dioica, Stellaria media, Geum urbanum,* and *Bromus sterilis*. Species that had more than 50% cumulative cover in the study plots were the following: *Bromus sterilis, Chelidonium majus, Poa nemoralis, Anthriscus sylvestris, Melica uniflora, Brachypodium sylvaticum, Urtica dioica, Poa pratensis, Stellaria holostea, Geum urbanum, Elymus repens, Anthriscus cerefolium, Rubus caesius, Humulus lupulus, Brachypodium pinnatum, Galium aparine,* and *Stellaria media*. The common characteristics of the most-mentioned species are the disturbance tolerance and nitrophily. However, the grass species characteristic of semi-natural forests of the study area (*Melica uniflora, Poa nemoralis, Brachypodium sylvaticum*) also play an essential role. The species composition is enriched with species like *Stachys sylvatica, Dryopteris filix-mas, Dryopteris carthusiana, Carex sylvatica, Carex pilosa, Sanicula europaea, Circaea lutetiana, Scrophularia nodosa*, and *Pulmonaria officinalis*, which represent the potential natural forest associations.

**Figure 1:**
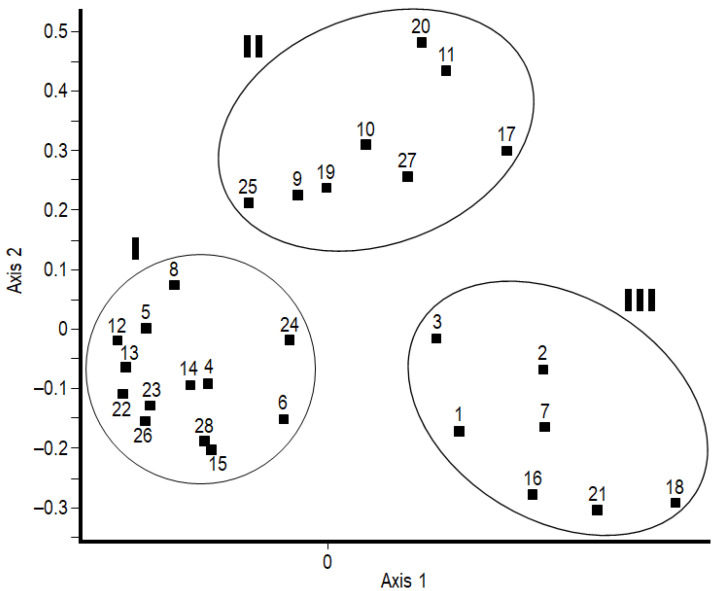
Principal coordinates analysis (PCoA) ordination of black locust stands based on the Bray–Curtis dissimilarity of the herb-layer.

**Figure 2:**
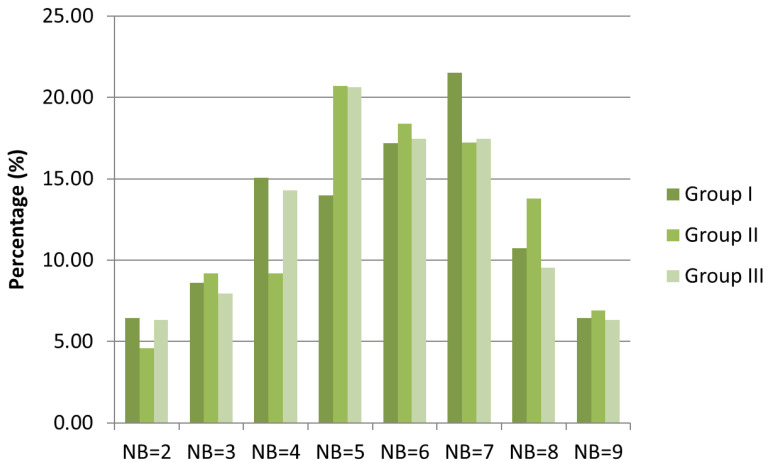
Numbers of species in the three groups separated via PCoA according to Borhidi’s nitrogen values (NB). Abbreviations for NB values: 1, plants only in soils extremely poor in mineral nitrogen; 2, plants of habitats very poor in nitrogen; 3, plants of moderately oligotrophic habitats; 4, plants of submesotrophic habitats; 5, plants of mesotrophic habitats; 6, plants of moderately nutrient-rich habitats; 7, plants of soils rich in mineral nitrogen; 8, N-indicator plants of fertilized soils; 9, plants only on hyperfertilized soil, extremely rich in nitrogen.

**Figure 3:**
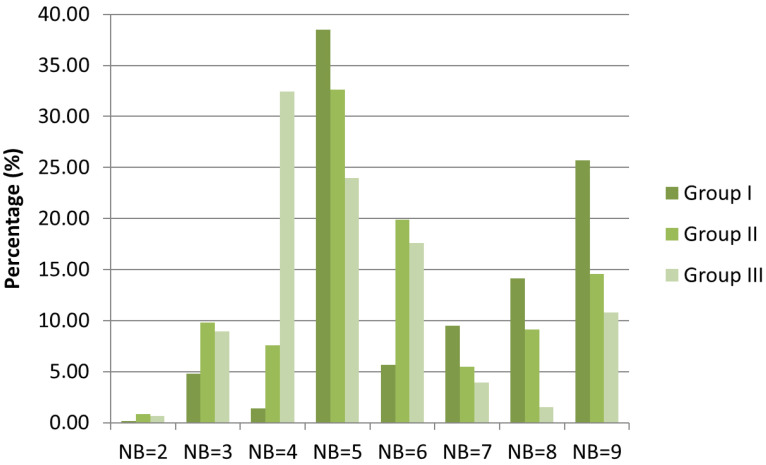
Covers of species in the three groups separated via PCoA according to Borhidi’s nitrogen values (NB). For NB values abbreviations, see Figure 2.

The principal coordinates analysis (PCoA) succeeded in separating three main groups, I-III (Figure 1). Based on the dominant structure of the herb-layer, the black locust stands in group I form a relatively uniform group (Figure 2) characterized by a high cover of typical nitrophilous species (NB: 8, 9). The most dominant species of this group were *Bromus sterilis* and *Chelidonium majus*, while further characteristic species were *Anthriscus sylvestris* and *Urtica dioica*. In some stands, *Poa pratensis, Elymus repens*, and *Poa nemoralis* also played an important role. The most important characteristic species of group II was *Brachypodium sylvaticum*. The cover of *Chelidonium majus* is also significant in this group, while in specific stands, species like *Poa nemoralis*, *P. pratensis*, and *Rubus caesius* also showed a substantial cover. Compared to group I, the overall floristic composition of group II was more similar to a sessile oak–Turkey oak forest, the potential forest community of the microregion. Notwithstanding, the cover of nitrophilous, weed, and disturbance-tolerant species was also significant (Figure 3). Black locust stands belonging to group III had an almost semi-natural herb-layer. The most characteristic species of this group III was *Melica uniflora*, accompanied with a high cover of *Stellaria holostea* in two stands only. Moreover, in some forest stands, the cover of *Urtica dioica* was also significant.

To examine the differences among the three statistically separated groups, we exemplified the distribution of Borhidi’s nitrogen values (NB) by the number and cover of species occurring in the herb-layer (Figure 2 and Figure 3). The distribution of species according to different nitrogen categories showed high similarity in the three groups. In the case of each group, the medium nitrophilous and moderately nitrophilous species (NB: 5, 6, 7) were the most prominent; hence, the presence of plants of habitats that were very poor in nitrogen and extremely rich in nitrogen (NB: 2, 9) reached very low values. The cover-weighted NB spectrum was better in pointing out the differences among the three groups. In group I, the cover of extremely nitrophilous species (NB: 8, 9) was much more significant than in groups II and III; however, the cover of mesotrophic plants was dominant. In group II, the plants of mesotrophic and moderately nutrient-rich habitats were the most significant. In addition, the plants of extremely nitrogen-rich habitats were also significant. In group III, the focus shifted to submesotrophic plants, although the cover of mesotrophic and moderately nitrophilous species was considerable.

### 2.2. Root-Nodule Formation

The number, surface area, and weight of root-nodules showed high variation, which differentiated the studied black locust stands well. In the case of three stands, no root-nodules were found, while the highest number of root-nodules per stand was 476. Between the two extremities, three orders of magnitude are represented; the number and the size of nodules were highly variable per stand (Table 1).

### 2.3. Root-Nodule and Herb-Layer Interactions

The Spearman’s rank correlations showed significant correlations between the covers of only 3 of the 17 studied dominant species (cumulative cover ≥ 50%) and root-nodule parameters, namely *Poa nemoralis, Geum urbanum,* and *Galium aparine*. The correlations were negative in all of the cases (Table 2). The three groups separated via PCoA on the basis of vegetation composition did not differ significantly on the basis of the number, surface area, and weight of the root-nodules (Kruskal–Wallis test, H = 0.865; 0.311; 0.457, ns). There was no significant difference between the root-nodule parameters (number, surface area, weight) of the sample sites with different exposures (Kruskal–Wallis test, H = 3.295; 4.402; 4.500, ns).

The principal component analysis (PCA) performed on the eight selected variables yielded three new independent variables (principal components) with eigenvalues > 1 that together explain 77.21% of the total variance (Table 3). With the exception of the cover of nitrophilous species, all habitat variables made a prominent contribution. The first component (PC1) accounted for 42.63% of the total variance, and it was highly and positively correlated with species richness, proportion of nitrophilous species, Shannon–Wiener diversity, and wood productivity.

The second component (PC2) accounted for an additional 21.75% of the total variance. This component was strongly and positively correlated with the herb-layer cover and negatively with the shrub layer cover. The third component (PC3), determined mainly by stand age, accounted for about 12.85% of the total variance.

Several significant relationships were obtained by investigating the correlations between the obtained PCA components and the root-nodule parameters (Table 4).

All root-nodule characteristics (number, surface area, and weight) were negatively associated with PCA1 and PCA3. At the same time, there was no significant relationship between the nodule parameters and PCA2, a component mainly determined by the herb-layer cover.

**Figure 4:**
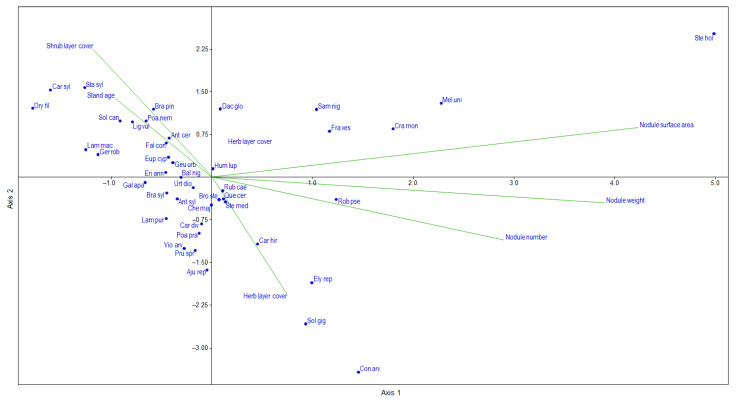
Ordination biplot from canonical correspondence analysis (CCA) of selected black locust stand variables and plant species. Plant species name abbreviations consist of the first three letters of the genus name followed by the first three letters of the species.

The canonical correspondence analysis (CCA) results allowed us to obtain more detailed information on the species-stand characteristics relationships (Figure 4). The eigenvalue of axes 1 and 2 were 0.191 and 0.154, respectively. The Monte Carlo permutation test confirmed the significance (*p* < 0.05) of the first two axes explaining the majority of variance (45.78% and 35.84%, respectively).

Among the variables included in the analysis, the first axis mainly represents the root-nodule parameters, while axis 2 primarily represents the cover of herb and shrub layers. Along axis 1, the separation of species with different nitrogen requirements can be partly observed. Typical nitrophilous species (e.g., *Robinia pseudoacacia*, *Sambucus nigra*) are mostly projected outwards from the origin of the positive side of axis 1. However, there are also some opposite examples, since plants of submesotrophic and mesotrophic habitats (e.g., *Stellaria holostea*, *Melica uniflora*) are also located in this direction. Perennial weed species (e.g., *Elymus repens*, *Solidago gigantea*, *Convolvulus arvensis*) are positioned on the negative side of axis 2, whereas predominantly silvicolous species (e.g., *Carex sylvatica*, *Stachys sylvatica*, *Dryopteris filix-mas*) are projected on the positive side of this same axis, along the stand age–shrub layer cover gradients.

## 3. Discussion

### 3.1. Herb-Layer Composition

Our studies proved that the most frequent and dominant species of studied black locust stands have relatively high nutrient and nitrogen demands, such as *Chelidonium majus, Urtica dioica, Stellaria media, Geum urbanum, Bromus sterilis, Anthriscus cerefolium*, and *Galium aparine*. Regarding the dominant component, the species composition showed remarkable similarity with other studies from different geographical regions, e.g., Hungary, east Austria, Czech Republic, South Poland, North Italy, and England [1,18,38,40,41,43], and even South Korea [39]. Nevertheless, our study showed a considerable presence of species of natural or semi-natural habitats, such as *Poa nemoralis, Melica uniflora*, and *Brachypodium sylvaticum*, similar to the sessile oak–Turkey oak forests, which occur only fragmentarily in the cultural landscape of the microregion of the study area [44]. Vítkova and Kolbek [40] also described plant communities (*Poa nemoralis-Robinietum, Melico transilvanicae-Robinietum*) with similar species composition and dominance of grass species from the Czech Republic. However, the species pool of studied black locust stands is significantly lower than the natural and semi-natural forest or grassland habitats of the region [45].

The principal coordinates analysis (PCoA) separated three main groups according to the herb-layer composition. These groups are differentiated according to the cover of species with different nitrogen demands. However, these groups did not differ significantly either on the basis of the geographical region or the root-nodule parameters. The species composition differences may be attributed to the different degrees of naturalness of the neighboring forest stands. Based on the results of canonical correspondence analysis (CCA), as expected, the perennial weed species were associated with the axis of herb-layer cover, while silvicolous species were associated with axis of stand age–shrub layer cover.

### 3.2. Root-Nodule Formation

The root-nodule parameters of the studied black locust stands differed greatly; there were stands without any root-nodules and stands with hundreds. The present study showed no significant differences between the root-nodule parameters of the study sites with different exposures. However, some studies highlighted the role of aspects in root-nodule formation. Noh et al. [34] found significant differences in the root-nodule biomass in north- and south-facing stands in central Korea. The root-nodule biomass was correlated to the soil temperature and water content; the south-facing stands with higher temperature and lower soil water content had considerably lower root-nodule biomass and mostly smaller root-nodules than the north-facing stands. On the contrary, some studies [46,47] demonstrated that because of drought, the drought stress increased the root-nodule biomass of black locust stands in order to maintain the appropriate nitrogen fixation rate in the case of lower soil nitrogen availability. Since our study sites were located in a hilly area with no significant differences in slope degrees, this factor probably did not play a role in the root-nodule formation. In addition to all of these factors, micro-topography can also play a major role in root-nodule formation, influencing solar radiation, soil temperature, water, and nutrient content [34,48,49,50]. As a result, there can be significant differences in the nodulation of individual trees, even within a stand, because of the small-scale heterogeneity of abiotic and biotic factors, so the general site characteristics may not always be suitable for estimating the root-nodule parameters of the whole stand.

### 3.3. Root-Nodule and Herb-Layer Interactions

Out of the seventeen dominant species, only in three cases was it possible to demonstrate a significant correlation with the root-nodule parameters. *Poa nemoralis, Geum urbanum*, and *Galium aparine* equally showed a negative correlation with root-nodule parameters. The canonical correspondence analysis (CCA) confirmed this contradictory relationship because the root-nodule parameters were similarly associated with nitrophilous, mesotrophic, and even submesotrophic species; therefore, it can be concluded that a clear connection between the vegetation and root-nodules could not be detected, and the species set in this case does not indicate root-nodule formation.

In the present study, all of the root-nodule characteristics (number, surface area, and weight) were negatively associated with PCA factor 1, mainly determined by species richness, cover and proportion of nitrophilous species, Shannon–Wiener diversity, and wood productivity. These results support the negative effect of black locust with nitrogen-fixing bacteria on biodiversity. However, some review reports highlight that the impact of species on invaded communities is ambiguous or contrasting [1,41]. The impact on biodiversity can depend on several factors, including the age and species composition of control stands, site environmental and biological characteristics, or even the means used for comparison. While Von Holle et al. [14] experienced significantly higher species richness and cover in black locust stands than in the native pine and mixed pine–oak stands, Rice et al. [13] found reduced native plant diversity in black locust stands when comparing the conservation value with native pine–oak ecosystems. Sitzia et al. [41] reported similar species richness and diversity of the understory plant species in black locust and native pioneer stands in the Eastern Alps. Akatov et al. [51] demonstrated similar alpha diversity in native riparian forests in Western Caucasus. The homogenizing and diversity-reducing effect of black locust was confirmed by Benesperi et al. [52] and Trentanovi et al. [53] in urban habitats in Berlin and in the Northern Apennines. Differences can also be manifested between black locust stands with different locations; Yang et al. [54] observed significantly lower species richness and Shannon–Wiener diversity of understory plants in tableland stands in contrast to gully stands on China’s Loess Plateau.

In our study, the nodule weight was negatively correlated with PCA factor 3 determined by stand age. This result is consistent with Boring and Swank’ study [32], which reported that the root-nodule biomass in different aged forest stands indicated that the peak of nitrogen fixation is characteristic from the early to intermediate stages of forest succession, and that the number of root-nodules is decreasing in the case of aging trees.

Several authors drew attention to the effect of soil characteristics on root-nodule formation, such as temperature, pH, dissolved organic carbon, and nitrate level [34,55,56]. Among these factors, the role of soil nitrogen is prominent; many studies verified that a high N supply in the soil limits the root-nodule formation and activity, not only by decreasing the nodule number and mass, but also by promoting nodule senescence [57,58,59,60]. However, this effect is not clearly manifested in all cases. Sun et al. [60] noted different responses in root-nodule development to changes in soil N availability in the case of black locust plants of different provenances but cultivated under the same greenhouse conditions. As the above-mentioned studies demonstrate, the connection between the soil parameters and root-nodule formation is diverse, mutually interacting, and sometimes contradictory. Therefore, in this study, we primarily examined whether the vegetation could indicate the root-nodule formulation, without examining the soil parameters.

## 4. Materials and Methods

### 4.1. Study Area

**Figure 5:**
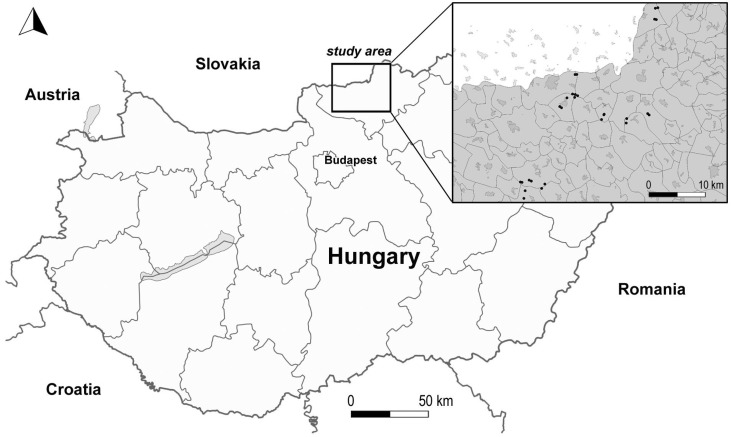
Study area and sites (black dots in the insert: forest subcompartments) in Cserhát Hills.

Our sample area is located in the Cserhát Hills (N47°55′, E19°30′), in the northern part of Hungary (Figure 5). It is characterized by a diverse and fragmented geological surface. The climatic conditions are moderately cold and moderately dry [61], with an annual precipitation of 560 to 620 mm/year, and an average annual temperature of 8–10 °C. According to the Hungarian forest climate classification [62], the dominant forest climate zone is the sessile oak–Turkey oak zone. A significant part of the study area is covered by forest plantations of non-indigenous tree species, mainly black locust.

The studied forest stands are found at elevations between 200 and 300 m above sea level (Table 5), and are dominated by brown forest soils (Cambisols and Podzols, according to the classification of IUSS Working Group [63]). Field studies were carried out in 28 randomly selected black locust forest subcompartments aged 3 to 32 years, providing a wide spectrum of site characteristics (e.g., stand age, exposure, slope).

### 4.2. Field Surveys and Laboratory Work

In each forest subcompartment, the elevation, exposure, slope, stand age, and wood productivity were recorded (Table 5). Wood productivity (m^3^/year/ha) was determined by the age and average height of the trees forming the stands [64].

During the botanical analysis, species and their cover were recorded in each layer (canopy, shrub, and herb) of every forest subcompartment. We used the following cover-abundance A-D scale according to the cover of different species: + = 0–1%, 1 = 1–5%, 2 = 5–25%, 3 = 25–50%, 4 = 50–75%, and 5 = 75–100% [65,66].

Soil samples were collected from the upper 20 cm layers according to the results of Boring [32] and Mantovani et al. [47], which showed the highest density of root-nodules in the upper 15 cm soil layer. We collected 24 soil samples of 8000 cm^3^ from each subcompartment. The 24 soil samples were composed of the following: three black locust trees selected randomly in each subcompartment, and eight soil samples collected near each sample tree. The samples were located in two circles; there were four samples next to the trunk and four samples at a 50–70 cm distance from it, depending on the canopy size. The soil sample cubes were separated into soil and root components. The root-nodules were counted and weighed without drying using an analytical balance (type KERN ABS 320-4N). Due to the approximately ellipsoidal shape of the root-nodules, their surface area was calculated using the Thomsen formula [67].

### 4.3. Data Analysis

To uncover differences in the floristic composition of the herb-layer of the studied forest stands, principal coordinates analysis (PCoA) based on the Bray–Curtis dissimilarity distance metric was performed with SYN-TAX 2000 software [68]. Statistically separated groups were characterized by Borhidi’s ecological indicator values (NB) for nitrogen [69], using the species composition and the cover of species. The nitrogen values were located on an ordinal scale (1–9) based on field observations (Figure 2), comparing the habitat preference and adaptation to nitrogen-rich habitats of the species [69].

The following parameters were included in the principal component analysis: stand age, wood productivity, species richness, Shannon–Wiener diversity, herb-layer cover, shrub layer cover, proportion of nitrophilous species, and cover of nitrophilous species. The proportion of nitrophilous species was determined by the percentage of nitrophilous species (species with NB 8, 9) compared to the total species number, while the cover of nitrophilous species was determined by the percent cover of nitrophilous species (species with NB 8, 9) compared to the cumulative cover of the occurring species.

To reduce the number of the variables determined for the studied forests, a principal component analysis (PCA) was performed using the software SPSS ver. 20.0 [70]. Prior to the PCA, the data were log-transformed to reduce their skewness [71]. Only PCA factors with eigenvalues exceeding 1.0 were selected (Kaiser criterion). The factor loadings were rotated with a varimax raw transformation. The obtained factors were tested for means using one-way ANOVA and post hoc Tukey’s test. The derived principal components were then correlated with the root-nodule parameters: number, surface area, and weight.

To explore potential relationships between plant species and black locust stand characteristics, canonical correspondence analysis (CCA) was applied as an ordination method [72] with forward variable selection. Prior to the analysis, the plant cover values were transformed using the Hellinger approach [73]. The forest environment matrix included the following variables: stand age, herb-layer cover, shrub layer cover, root-nodule number, root-nodule surface area, and root-nodule weight. Plant species occurring in less than five sites and/or species with less than 5% coverage were not entered in the analysis to avoid misrepresentation of the survey data. An unrestricted Monte Carlo test with 1000 randomizations was performed in parallel to determine the significance of the axes. The analyses were carried out using the software Past ver. 4.03 [74].

The correlation between the cover of dominant species (cumulative cover ≥ 50%) and root-nodule parameters was tested using Spearman’s rank correlation. The Kruskal–Wallis test was used to compare root-nodule characteristics among the groups separated by the PCoA based on vegetation composition, and among sites with different exposures.

## 5. Conclusions

The studied black locust stands showed high variability both in their herb-layer compositions and root-nodule formation, but no clear relationship could be demonstrated between these characteristics. Only the covers of three species (*Poa nemoralis, Geum urbanum,* and *Galium aparine*) showed a significant correlation with root-nodule parameters. This correlation was negative in all cases, despite the fact that these species can be characterized by different nitrogen demands. Therefore, it can be concluded that the herb-layer species do not clearly indicate the root-nodule formation in this study. In addition, the PCA component with which the species richness and Shannon–Wiener diversity were strongly correlated was negatively associated with all root-nodule parameters (number, surface area, and weight), supporting the biodiversity-reducing effect of black locust by its nitrogen-fixing bacteria. All root-nodule parameters were negatively correlated with the PCA factor predominantly determined by stand age, confirming that the root-nodule biomass decreases as time progresses.

As the literature review shows, root-nodule formation is influenced by several mutually interacting factors, including a site’s abiotic and biotic characteristics, and even the individual differences in the adaptive capacity of black locust. It is difficult to interpret these diverse interactions in nature; therefore, every study’s findings are essential, which help to understand the nitrogen dynamics in forest ecosystems [34]. Several authors [23,34,60,75] emphasize the great need for further detailed studies about the effects of N_2_-fixing woody legumes, as they are underrepresented compared to the number of similar studies on herbaceous plants. Since the black locust is a species of high importance worldwide through its considerable ecological and economic effects, which cover a wide range of uses from tree cultivation to ornamental use [34,76,77], we hope that we can contribute to the understanding of the diverse interactions of this species and provide new starting points for further studies.

## Figures and Tables

**Table 1:** Herb-layer and root-nodule characteristics (mean ± SE) of the studied forest subcompartments (n = 28).

Characteristics	Min	Max	Mean	SE
Species richness	8	40	21.25	1.48
Shrub layer cover (%)	5	80	42.46	3.84
Herb-layer cover (%)	38	100	78.32	3.92
Shannon–Wiener diversity	1	2.27	1.71	0.07
Proportion of nitrophilous species (%)	0.15	0.58	0.30	0.02
Cover of nitrophilous species (%)	1	88	39.29	5.30
Number of root-nodules (Nr.)	0	476	116.61	26.00
Surface area of root-nodules (mm^2^)	0	10,487	1196.79	394.46
Weight of root-nodules (g)	0	4.84	0.69	0.22

**Table 2:** Spearman’s rank correlations between the cover of dominant species and root-nodule characteristics. Significant correlations are highlighted in bold.

Species	Nodule Number		Nodule Surface Area		Nodule Weight	
	*r*	*p*	*r*	*p*	*r*	*p*
*Bromus sterilis*	−0.099	0.615	0.009	0.963	−0.063	0.749
*Chelidonium majus*	−0.109	0.581	−0.027	0.892	−0.041	0.838
*Poa nemoralis*	**−0.438**	**0.020**	**−0.413**	**0.029**	**−0.388**	**0.041**
*Anthriscus sylvestris*	−0.239	0.220	−0.247	0.205	−0.242	0.214
*Melica uniflora*	0.062	0.753	−0.031	0.875	−0.065	0.743
*Brachypodium sylvaticum*	−0.196	0.317	−0.266	0.171	−0.315	0.103
*Urtica dioica*	−0.242	0.215	−0.355	0.064	−0.343	0.074
*Poa pratensis*	−0.332	0.085	−0.244	0.212	−0.312	0.106
*Stellaria holostea*	0.253	0.194	0.163	0.408	0.136	0.490
*Geum urbanum*	−0.324	0.092	**−0.407**	**0.031**	**−0.381**	**0.045**
*Elymus repens*	0.053	0.790	0.167	0.396	0.139	0.482
*Anthriscus cerefolium*	−0.172	0.381	−0.108	0.584	−0.111	0.575
*Rubus caesius*	0.096	0.628	0.092	0.643	0.080	0.687
*Humulus lupulus*	−0.052	0.792	−0.061	0.759	−0.039	0.845
*Brachypodium pinnatum*	−0.300	0.121	−0.240	0.219	−0.253	0.194
*Galium aparine*	**−0.479**	**0.010**	**−0.381**	**0.046**	**−0.369**	**0.053**
*Stellaria media*	−0.036	0.858	0.006	0.976	−0.007	0.974

**Table 3:** Factor loadings for the first three principal components in PCA on the black locust stand variables used. The values of variables that are determinants for a given PC are highlighted in bold.

Stand Variables	PC1	PC2	PC3
Species richness	**0.770**	0.446	−0.111
Cover of nitrophilous species	**0.926**	0.198	0.064
Proportion of nitrophilous species	**0.708**	−0.306	0.364
Shannon–Wiener diversity	**0.812**	0.146	−0.185
Herb-layer cover	0.150	**−0.846**	−0.170
Shrub layer cover	0.169	**0.818**	−0.010
Stand age	0.044	0.127	**0.909**
Wood productivity	**0.831**	0.005	0.177
Eigenvalue	3.410	1.740	1.028
Explained variance %	42.63	21.75	12.85
Cumulated variance %	42.63	64.38	77.21

**Table 4:** Correlations between the PCA components and root-nodule characteristics. Significant correlations are highlighted in bold.

Nodule Characteristics	PC1			PC2			PC3		
	*r*	*F*	*p*	*r*	*F*	*p*	*r*	*F*	*p*
Nodule number	−0.556	11.66	**0.002**	0.042	0.046	0.833	−0.403	5.056	**0.033**
Nodule surface area	−0.662	20.315	**0.0001**	−0.113	0.339	0.566	−0.454	3.660	**0.015**
Nodule weight	−0.601	14.691	**0.0007**	−0.091	0.329	0.644	−0.514	9.320	**0.005**

**Table 5:** Site characteristics of forest subcompartments.

Site (Forest Subcompartments)	Elevation (m)	Exposure	Slope (°)	Stand Age (year)	Wood Productivity (m^3^/year/ha)
Alsópetény 29D	200	S	20	19	8
Alsópetény 7F	200	S	15	23	11
Iliny 1A	200	N	15	15	14
Iliny 1B	200	S	15	22	13
Nógrádmarcal 1A	200	N	25	20	13
Nógrádmarcal 1D	200	S	15	20	10
Nógrádmarcal 9C	200	N	10	32	10
Nógrádszakál 12G	300	S	15	12	8
Nógrádszakál 12I	200	S	10	8	8
Nógrádszakál 5C	300	W	10	26	10
Nógrádszakál 5D	300	E	10	25	10
Patvarc 1A	150	N	5	21	11
Patvarc 1B	150	*	0	24	10
Rimóc 4 B	300	S	20	18	9
Rimóc 4G	300	S	15	8	13
Romhány 12A	200	*	15	9	8
Romhány 1E	300	N	15	25	7
Romhány 21D	300	N	10	8	4
Romhány 22C	300	N	10	15	8
Romhány 4B	300	N	10	9	7
Romhány 8C	200	*	10	28	8
Szügy 25B	200	*	0	21	12
Szügy 25D	200	*	0	3	11
Szügy 2D	200	W	10	27	10
Szügy 3B	200	*	15	8	15
Szügy 5G	200	N	5	14	9
Varsány 14C	200	*	10	17	13
Varsány 17I	200	S	10	29	8

* clear exposure cannot be determined (plain or variable).

## Data Availability

The data presented in this study are available on request from the corresponding author. The data are not publicly available due to the data management policy of the local forest management company Ipoly Erdő LC.

## Supplementary Materials

The following supporting information can be downloaded at: https://www.mdpi.com/article/10.3390/plants12183253/s1, Table S1: The species occurring in herb-layer in the order of cumulative cover.
