# Supplementary material for: Diverse Interactions: Root-Nodule Formation and Herb-Layer Composition in Black Locust (Robinia pseudoacacia) Stands
Source: Plants (Basel). 2023 Sep 13;12(18):3253. doi: 10.3390/plants12183253 (PMC10534852; doi:10.3390/plants12183253)
Supplement: Supplementary file 1 [file plants-12-03253-s001.zip › plants-2553142-supplementary.pdf]

Supplementary material

Table S1. The species occurring in herb layer in the order of cumulative cover

Species	Cumulative cover
Bromus sterilis	631.0
Chelidonium majus	352.5
Melica uniflora	266.0
Poa nemoralis	242.5
Brachypodium sylvaticum	213.0
Anthriscus sylvestris	209.5
Urtica dioica	192.0
Poa pratensis	143.0
Stellaria holostea	103.5
Geum urbanum	83.0
Elymus repens	73.5
Anthriscus cerefolium	62.5
Rubus caesius	62.5
Humulus lupulus	58.5
Brachypodium pinnatum	55.5
Galium aparine	54.5
Stellaria media	50.0
Ballota nigra	46.5
Convolvulus arvensis	41.5
Lamium purpureum	39.0
Lamium maculatum	37.5
Ajuga reptans	34.0
Solidago canadensis	33.0
Stachys sylvatica	24.0
Dryopteris filix-mas	19.0
Carex sylvatica	18.0
Ligustrum vulgare	17.0
Cornus sanguinea	16.0
Carex pilosa	15.0
Fallopia convolvulus	12.5
Carex hirta	8.5
Crataegus monogyna	8.0
Erigeron annuus	8.0
Geranium robertianum	7.5
Robinia pseudoacacia	7.5
Solidago gigantea	6.0
Carex divulsa	5.5
Dactylis glomerata	5.5
Euphorbia cyparissias	5.5
Fragaria vesca	5.0
Galeobdolon luteum	5.0
Prunus spinosa	5.0
Quercus cerris	5.0
Sambucus ebulus	5.0

Galeopsis pubescens	4.5
Sambucus nigra	4.5
Viola arvensis	4.5
Calamagrostis epigeios	4.0
Dryopteris carthusiana	4.0
Clematis vitalba	3.0
Festuca rupicola	3.0
Galeopsis speciosa	3.0
Hypericum perforatum	3.0
Acer campestre	2.5
Arctium lappa	2.5
Fragaria viridis	2.5
Alliaria petiolata	2.0
Mycelis muralis	2.0
Sanicula europaea	2.0
Silene latifolia	2.0
Viola reichenbachiana	2.0
Agrimonia eupatoria	1.5
Carex remota	1.5
Carpinus betulus	1.5
Chenopodium album	1.5
Erigeron canadensis	1.5
Juncus effusus	1.5
Physalis alkekengi	1.5
Quercus petraea	1.5
Rosa canina	1.5
Setaria pumila	1.5
Acer platanoides	1.0
Alopecurus pratensis	1.0
Asclepias syriaca	1.0
Cannabis sativa	1.0
Circaea lutetiana	1.0
Cornus mas	1.0
Leonurus cardiaca	1.0
Polygonatum multiflorum	1.0
Polygonum lapathifolium	1.0
Polygonum persicaria	1.0
Prunella vulgaris	1.0
Rumex sanguineus	1.0
Veronica hederifolia	1.0
Ambrosia artemisifolia	0.5
Arrhenatherum elatius	0.5
Asparagus officinalis	0.5
Astragalus glycyphyllos	0.5
Berteroa incana	0.5
Bidens tripartitus	0.5
Bromus inermis	0.5
Calystegia sepium	0.5
Campanula trachelium	0.5
Cardamine impatiens	0.5

Celtis occidentalis	0.5
Clinopodium vulgare	0.5
Conium maculatum	0.5
Corylus avellana	0.5
Crataegus laevigata	0.5
Cruciata laevipes	0.5
Cydonia oblonga	0.5
Echinochloa crus-galli	0.5
Fagus sylvatica	0.5
Glechoma hederacea	0.5
Ornithogalum boucheanum	0.5
Parietaria officinalis	0.5
Plantago major	0.5
Prunus padus	0.5
Pulmonaria officinalis	0.5
Pyrus pyraster	0.5
Quercus robur	0.5
Rumex acetosella	0.5
Salix alba	0.5
Saponaria officinalis	0.5
Scrophularia nodosa	0.5
Setaria viridis	0.5
Silene noctiflora	0.5
Taraxacum officinale	0.5
Ulmus minor	0.5
Verbascum phoeniceum	0.5
Viola hirta	0.5
